# Structural effects of sprifermin in knee osteoarthritis: a post-hoc analysis on cartilage and non-cartilaginous tissue alterations in a randomized controlled trial

**DOI:** 10.1186/s12891-016-1128-2

**Published:** 2016-07-09

**Authors:** Frank W. Roemer, Aida Aydemir, Stefan Lohmander, Michel D. Crema, Monica Dias Marra, Norma Muurahainen, David T. Felson, Felix Eckstein, Ali Guermazi

**Affiliations:** Quantitative Imaging Center (QIC), Boston University School of Medicine, Boston, MA USA; Department of Radiology, University of Erlangen-Nuremberg, Erlangen, Germany; EMD Serono, Billerica, MA USA; Department of Orthopaedics, Lund University, Lund, Sweden; Clinical Epidemiology Research and Training Unit, Boston University School of Medicine, Boston, MA USA; Department of Anatomy, Paracelsus Medical University, Salzburg, Austria; Chondometrics GmbH, Ainring, Germany

**Keywords:** Magnetic resonance imaging, Osteoarthritis, Cartilage, Bone marrow lesions, Sprifermin

## Abstract

**Background:**

A recent publication on efficacy of Sprifermin for knee osteoarthritis (OA) using quantitatively MRI-defined central medial tibio-femoral compartment cartilage thickness as the structural primary endpoint reported no statistically significant dose response. However, Sprifermin was associated with statistically significant, dose-dependent reductions in loss of total and lateral tibio-femoral cartilage thickness. Based on these preliminary promising data a post-hoc analysis of secondary assessment and endpoints was performed to evaluate potential effects of Sprifermin on semi-quantitatively evaluated structural MRI parameters. Aim of the present analysis was to determine effects of sprifermin on several knee joint tissues over a 12 month period.

**Methods:**

1.5 T or 3 T MRIs were acquired at baseline and 12 months follow-up using a standard protocol. MRIs were read according to the Whole-Organ Magnetic Resonance Imaging Score (WORMS) scoring system (in 14 articular subregions) by four muskuloskeletal radiologists independently. Analyses focused on semiquantitative changes in the 100 μg subgroup and matching placebo of multiple MRI-defined structural alterations. Analyses included a delta-subregional and delta-sum approach for the whole knee and the medial and lateral tibio-femoral (MTFJ, LTFJ), and patello-femoral (PFJ) compartments, taking into account number of subregions showing no change, improvement or worsening and changes in the sum of subregional scores. Mann–Whitney − Wilcoxon tests assessed differences between groups.

**Results:**

Fifty-seven and 18 patients were included in the treatment and matched placebo subgroups. Less worsening of cartilage damage was observed from baseline to 12 months in the PFJ (0.02, 95 % confidence interval (CI) (−0.04, 0.08) vs. placebo 0.22, 95 % CI (−0.05, 0.49), *p* = 0.046). For bone marrow lesions (BMLs), more improvement was observed from 6 to 12 months for whole knee analyses (−0.14, 95 % CI (−0.48, 0.19) vs. placebo 0.44, 95 % CI (−0.15, 1.04), *p* = 0.042) although no significant effects were seen from the baseline visit, or in Hoffa-synovitis, effusion-synovitis, menisci and osteophytes.

**Conclusions:**

In this post-hoc analysis cartilage showed less worsening from baseline to 12 months in the PFJ, and BMLs showed more improvement from 6 to 12 months for the whole knee.

**Trial registration:**

ClinicalTrials.gov identifier: NCT01033994.

## Background

The treatment of knee OA is currently restricted to management of pain and function with low-to-moderate efficacy. The potential of a combined beneficial effect on joint structure and symptoms in OA as a result of treatment with several pharmacologic compounds has been investigated [1–4]. However, none of these agents has been shown to have unequivocally beneficial effects on structural characteristics that also translate into clinical benefit [5]. Many studies have focused on use of anticatabolic agents to delay progression [5]. An alternative approach is to stimulate cartilage development and repair. Sprifermin (recombinant human fibroblast growth factor 18; rhFGF18) binds to and specifically activates fibroblast growth factor receptor 3 (FGFR-3) in cartilage to promote chondrogenesis and cartilage matrix production in vitro [6]. Preclinical data have shown that sprifermin stimulates chondrocyte proliferation, cartilage matrix formation, and cartilage repair in vitro and in vivo [6, 7]. Recently, the results of a randomized, double-blind, placebo-controlled phase 1b clinical trial of sprifermin in knee OA were reported [8]. In that trial, no statistically significant response was seen in the primary structural endpoint, the central medial tibio-femoral compartment cartilage thickness on quantitative MRI (qMRI) [8]. However, pre-specified secondary structural efficacy endpoints, such as loss of total and lateral tibio-femoral cartilage thickness, showed statistically significant dose-dependent effects. Given the close interaction of the different tissues on a local level within the joint and its relevance to structural progression [9], changes in cartilage surface morphology may also be associated with an impact on the subchondral bone and other joint structures.

For these reasons a post-hoc investigation taking into account changes in multiple joint tissues based on semi-quantitative scoring and using advanced analytic approaches was conducted. The aim of this post-hoc analysis was to determine effects of sprifermin on semi-quantitative MRI (sqMRI) features of knee OA compared with placebo treatment.

## Methods

### Study design

Details of study design and patient inclusion have been reported [8]. In brief, in this multicenter, randomized, double blind, placebo-controlled trial, (ClinicalTrials.gov identifier: NCT01033994), sprifermin was evaluated as a single treatment and as a multiple-dose regimen with three doses of either 10 μg, 30 μg, or 100 μg with 21, 42 and 63 patients respectively, and matched placebo groups of 7, 14 and 21 patients, respectively. Patients were aged ≥40 years, had an established diagnosis of primary tibio-femoral knee OA according to American College of Rheumatology clinical and radiologic criteria, with Kellgren-Lawrence (KL) grade 2 or 3 disease in the target knee [10]. Altogether 477 patients were screened and 192 were randomized (24 to the single-dose cohorts and 168 to the multiple-dose cohorts. All patients in the single-dose cohorts received treatment with the study drug and completed the trial. All patients in the multiple-dose cohorts received ≥1 dose of study medication, with 168 forming the modified intent-to-treat population; 156 (92.9 %) completed the trial.

**Fig. 1:**
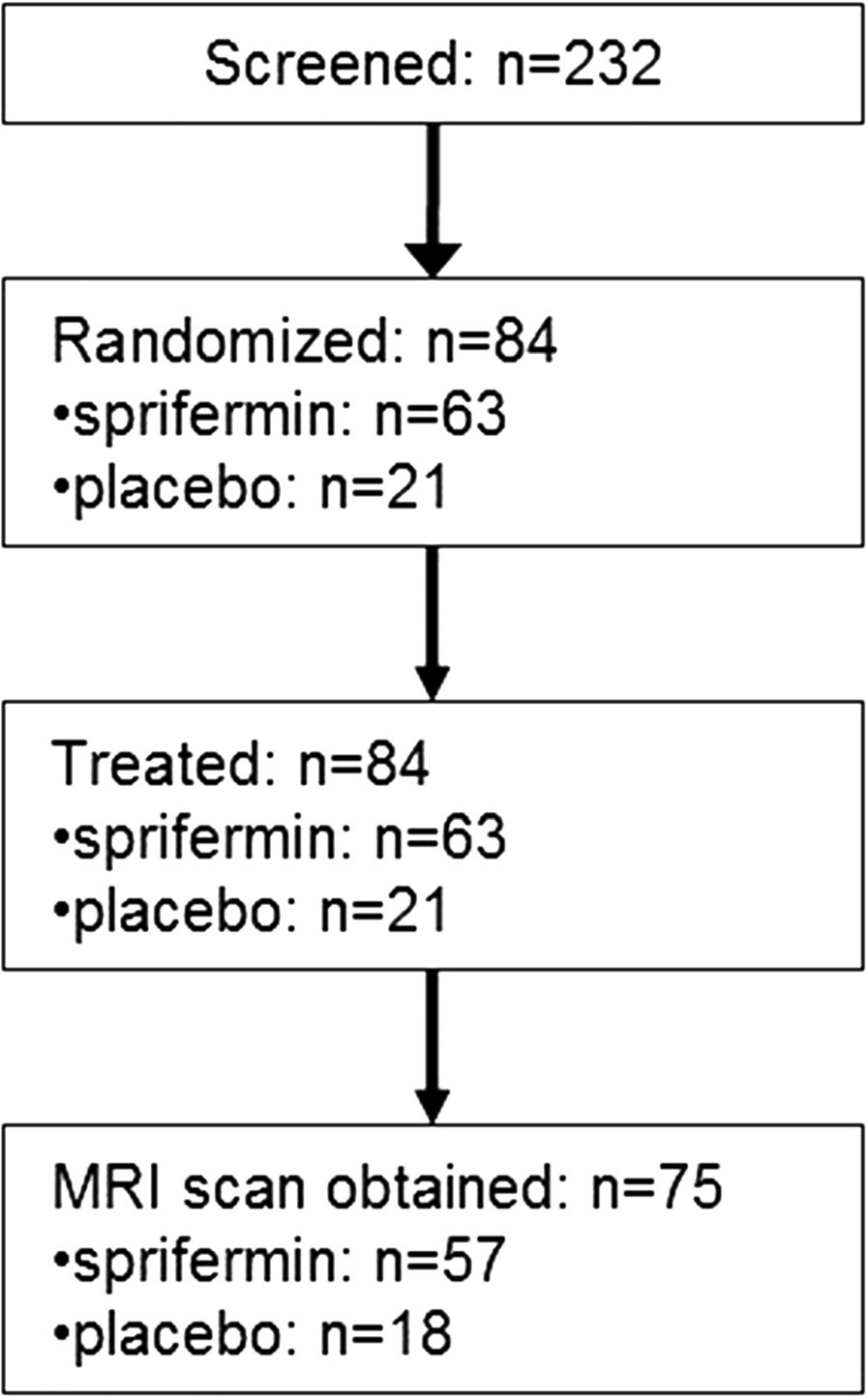
Detailed flow-chart of subject inclusion to the 100 μg subgroup

Similar to the recently reported post-hoc analysis on quantitative cartilage parameters [11], the current sqMRI analysis focuses on subjects with baseline and 12 month data in the cohort that received sprifermin 100 μg (*n* = 57) (since this was the dosing regimen on which significant drug efficacy was observed [8]), and in those who were randomized, in parallel, to receive matching placebo (*n* = 18). Since this multicenter trial enrolled cohorts sequentially using a dose-ascending approach across 30 sites on multiple continents [11], a comparison of the 100 μg subgroup with a combined placebo subgroup incorporating all dosages (*n* = 42) was not warranted. The detailed flow-chart of inclusion of the current analysis is presented in Fig. 1.

### Primary end points and assessments

The primary efficacy end point was the longitudinal change from baseline in central medial tibio-femoral compartment cartilage thickness at 6 months and 12 months, as assessed using qMRI [8].

### Secondary end points and assessments

Secondary imaging end points included total and compartmental femorotibial cartilage thickness and volume as assessed by qMRI, measurement of joint space width by fixed-flexion weight-bearing radiography, and assessment of bone marrow lesions (BMLs), cartilage, menisci, osteophytes, effusion, and synovitis by sqMRI using the modified Whole-Organ Magnetic Resonance Imaging Score (WORMS) at baseline, 3, 6, and 12 months follow-up [12].

### MRI acquisition

MRIs were acquired using 1.5 T or 3 T systems. Axial, coronal, and sagittal intermediate-weighted turbo or fast spin–echo fat-suppressed sequences were used for semiquantitative whole joint assessment of structural tissue pathology, with identical parameters, software and hardware used at baseline and follow-up. Image parameters were as follows: slice thickness 3.0 mm, in-plane resolution 0.55 × 0.55 mm, repetition time 3,600–4,000 msec, and echo time 30–40 msec. In addition the double-oblique coronal spoiled gradient-recalled sequences with fat suppression or water excitation that were acquired for cartilage thickness determination using quantitative MRI were considered for sqMRI assessment with the following acquisition paramaters: contiguous slice thickness 1.5 mm, in-plane resolutions 0.23 × 0.23 mm to 0.32 × 0.32 mm, repetition time 18–50 msec, echo time 6.5–14 msec, flip angle 15–20°.

### MRI interpretation

Four musculoskeletal radiologists (FWR, AG, MDC, MDM), with 7–16 years experience in standardized sqMRI assessment of knee OA, graded cartilage status, BMLs, osteophytes, effusion, synovitis and meniscal morphology according to the WORMS system [12] with blinding to treatment, radiographic OA grade and clinical data. In addition to the published WORMS scale medial and lateral meniscal extrusion was assessed on the coronal plane according to previous publications [13, 14] and graded as follows: 0 = no meniscal extrusion,1 = extrusion < 50 %, 2 = extrusion ≥ 50 %. Baseline and follow-up MRIs were presented sequentially, with the chronological acquisition order known to the readers. The four readers assessed the images independently with an equal number of examinations assigned to each reader.

### Statistical analysis

Statistical analyses were performed using SAS software (version 9.1; SAS Institute). Included in the current analysis were patients from the 100 μg and matched placebo subgroups with complete MRI datasets with all features and subregions scorable. Analyses included multi-dimensional assessment: (a) A delta-subregional approach was applied, which adds the *number of subregions* (total of 14 articular subregions for cartilage and BMLs on a knee level, 5 subregions each for the medial and lateral tibio-femoral [MTFJ, LTFJ] and 4 subregions for the patello-femoral [PFJ] compartment) showing worsening (>0), no change (0) or improvement (<0). As an example, 5 subregions showing worsening, 7 subregions showing improvement and 3 subregions showing no change will result in a delta-subregion change of −2. (b) In addition, a delta-sum approach was used, which adds the absolute *scores of all subregions* combined per compartment or for the whole knee. Analyses were performed on a whole knee level and separately for MTFJ, LTFJ and PFJ compartments for all subgroups. Mann–Whitney − Wilcoxon tests assessed differences between treatment groups. In sensitivity analysis adjusting for baseline values, analysis of covariance (ANCOVA) on ranked baseline and post baseline values was performed. *P*-values were not adjusted for multiple testing.

## Results

In regard to baseline demographic characteristics the 100 μg and matched placebo subgroup were comparable without statistically significant differences in regard to age, gender, BMI, KL grade and time since onset of OA. The detailed patient demographics are presented in Table 1.

**Table 1:** Patient demographics

	100 μg group (*n* = 57)	Matched placebo group (*n* = 18)
Age, mean (standard deviation)	61.2 (9.1)	60.9 (6.9)
Sex, no. (%) female	39 (68.4)	12 (66.7)
Body mass index (standard deviation)	30.5 (5.0)	31.5 (5.3)
Kellgren-Lawrence grade 3 (%)	29 (50.9)	11 (61.1)
Time since onset of osteoarthritis, years (standard deviation)	7.1 (5.4)	7.4 (3.7)

For change in cartilage surface morphology from baseline to 12 months, statistically significant differences with less worsening in the treatment group were observed in the PFJ (delta-sum approach; treatment 0.05, 95 % CI (−0.06, 0.17) vs. placebo 0.44, 95 % CI (−0.18, 1.06), *p* = 0.048; delta-subregional approach; treatment (0.02 95 % CI (−0.04, 0.08) vs. placebo 0.22, 95 % CI (−0.05, 0.49), *p* = 0.046). Although baseline values were statistically different, after adjusting for baseline in ANCOVA sensitivity analysis, results remained statistically significant at a significance level of 0.05.

For change in BMLs from baseline to 12 months only, no significant findings were seen for either treatment or placebo groups using both analytic approaches.

The details of the baseline to 12 months analyses for cartilage and BMLs are presented in Table 2.

**Table 2:** Cartilage morphology and bone marrow lesion ─ baseline and change from baseline to 12 months

		Cartilage morphology			Bone marrow lesion		
		100 μg cohort (*n* = 57) mean (95 % CI)	Matched placebo (*n* = 18) mean (95 % CI)	*p* - value	100 μg cohort (*n* = 56) mean (95 % CI)	Matched placebo (*n* = 18) mean (95 % CI)	*p* - value
Whole knee	Baseline	21.1 (17.2, 25.0)	30.0 (22.0,37.9)	0.040*	3.3 (2.5, 4.1)	3.3 (1.4, 5.2)	0.642
	Delta sum	0.42 (0.00, 0.84)	0.94 (−0.34, 2.23)	0.362	−0.20 (−0.67, 0.28)	0.22 (−0.62, 1.07)	0.237
	Delta subregion	0.19 (−0.01, 0.40)	0.44 (−0.24, 1.13)	0.371	−0.23 (−0.60, 0.14)	0.22 (−0.43, 0.87)	0.113
LTFJ	Baseline	4.5 (2.6, 6.4)	7.6 (3.8, 11.5)	0.019*	0.7 (0.3, 1.1)	0.7 (0.0, 1.4)	0.981
	Delta sum	0.07 (−0.16, 0.30)	0.17 (−0.09, 0.42)	0.342	0.04 (−0.16, 0.23)	0.11 (−0.05, 0.27)	0.242
	Delta subregion	0.05 (−0.07, 0.18)	0.11 (−0.05, 0.27)	0.349	0.04 (−0.13, 0.20)	0.17 (−0.02, 0.36)	0.107
MTFJ	Baseline	9.3 (7.2, 11.5)	11.5 (7.3, 15.8)	0.352	1.6 (1.1, 2.2)	1.1 (0.3, 1.9)	0.418
	Delta sum	0.30 (−0.04, 0.64)	0.33 (−0.28, 0.95)	0.690	−0.11 (−0.50, 0.28)	0.11 (−0.52, 0.75)	0.469
	Delta subregion	0.12 (−0.03, 0.27)	0.11 (−0.27, 0.49)	0.712	−0.13 (−0.40, 0.15)	0.06 (−0.34, 0.45)	0.510
PFJ	Baseline	7.3 (5.6., 9.1)	10.8 (7.5, 14.1)	0.038*	1.0 (0.5, 1.5)	1.5 (0.1, 2.9)	1.000
	Delta sum	0.05 (−0.06, 0.17)	0.44 (−0.18, 1.06)	0.048*	−0.13 (−0.34, 0.09)	0.00 (−0.38, 0.38)	0.368
	Delta subregion	0.02 (−0.04, 0.08)	0.22 (−0.05, 0.49)	0.046*	−0.14 (−0.34, 0.06)	0.00 (−0.38, 0.38)	0.352

*CI* confidence interval, *LTFJ* lateral tibio-femoral joint (includes 5 subregions), *MTFJ* medial tibio-femoral joint (includes 5 subregions), *PFJ* patello-femoral joint (includes 4 subregions). Possible score range for each subregion at baseline and 12-month is 0–3 and 0–6 for bone marrow lesion and cartilage morphology, respectively

*Statistically significant at *p* ≤ 0.05

For BMLs, in post-hoc evaluation, all interim visits were analyzed in a non-preplanned time frame. Statistically significant changes in BMLs in favor of the treatment group were observed for the whole knee from 6 to 12 months (delta-subregional approach; treatment −0.14, 95 % CI (−0.48, 0.19) vs. placebo 0.44, 95 % CI (−0.15, 1.04), *p* = 0.042), and were borderline for the LTFJ (delta-sum approach; treatment −0.13, 95 % CI (−0.39, 0.14) vs. placebo 0.22, 95 % CI (−0.10, 0.54), *p* = 0.062; delta-subregion approach: −0.05, 95 % CI (−0.27, 0.16) vs placebo 0.22, 95 % CI (−0.10, 0.54), *p* = 0.064). The detailed BML analyses for the different intervals are presented in Table 3. For all other analyzed parameters including meniscus, osteophytes, effusion-synovitis and Hoffa-synovitis, no significant changes from baseline to 12 months or between groups were observed.

**Table 3:** Bone marrow lesion changes between interim visits

		100 μg cohort (*n* = 56) Mean (95 % CI)	Matched placebo (*n* = 18) Mean (95 % CI)	*p* - value
Whole knee	Baseline	3.3 (2.5, 4.1)	3.3 (1.4, 5.2)	0.642
	Delta sum			
	0 to 3 months	−0.02 (−0.38, 0.34)	−0.17 (−0.85, 0.52)	0.682
	3 to 6 months	−0.04 (−0.41, 0.34)	−0.06 (−0.37, 0.26)	0.933
	6 to 12 months	−0.14 (−0.54, 0.26)	0.44 (−0.15, 1.04)	0.054
	Delta subregion			
	0 to 3 months	0.02 (−0.28, 0.32)	−0.22 (−0.87, 0.43)	0.597
	3 to 6 months	−0.05 (−0.37, 0.27)	−0.06 (−0.37, 0.26)	0.911
	6 to 12 months	−0.14 (−0.48, 0.19)	0.44 (−0.15, 1.04)	0.042*
LTFJ	Baseline	0.7 (0.3, 1.1)	0.7 (0.0, 1.4)	0.981
	Delta sum			
	0 to 3 months	0.04 (−0.12, 0.19)	0.00 (−0.17, 0.17)	0.859
	3 to 6 months	0.13 (−0.11, 0.36)	−0.11 (−0.40, 0.18)	0.155
	6 to 12 months	−0.13 (−0.39, 0.14)	0.22 (−0.10, 0.54)	0.062
	Delta subregion			
	0 to 3 months	0.04 (−0.12, 0.19)	0.00 (−0.17, 0.17)	0.859
	3 to 6 months	0.09 (−0.12, 0.30)	−0.11 (−0.40, 0.18)	0.155
	6 to 12 months	−0.05 (−0.27, 0.16)	0.22 (−0.10, 0.54)	0.064
MTFJ	Baseline	1.6 (1.1, 2.2)	1.1 (0.3, 1.9)	0.418
	Delta sum			
	0 to 3 months	−0.11 (−0.41, 0.20)	−0.11 (−0.49, 0.27)	0.727
	3 to 6 months	−0.07 (−0.27, 0.13)	0.06 (−0.21, 0.32)	0.530
	6 to 12 months	0.07 (−0.21, 0.35)	0.17 (−0.38, 0.71)	0.673
	Delta subregion			
	0 to 3 months	−0.07 (−0.30, 0.16)	−0.17 (−0.47, 0.14)	0.574
	3 to 6 months	−0.05 (−0.21, 0.10)	0.06 (−0.21, 0.32)	0.525
	6 to 12 months	0.02 (−0.20, 0.24)	0.17 (−0.38, 0.71)	0.646
PFJ	Baseline	1.0 (0.5, 1.5)	1.5 (0.1, 2.9)	1.000
	Delta sum			
	0 to 3 months	0.05 (−0.09, 0.20)	−0.06 (−0.45, 0.34)	0.772
	3 to 6 months	−0.09 (−0.24, 0.06)	0.00 (−0.24, 0.24)	0.647
	6 to 12 months	−0.09 (−0.31, 0.13)	0.06 (−0.38, 0.49)	0.123
	Delta subregion			
	0 to 3 months	0.05 (−0.09, 0.20)	−0.06 (−0.45, 0.34)	0.772
	3 to 6 months	−0.09 (−0.24, 0.06)	0.00 (−0.24, 0.24)	0.647
	6 to 12 months	−0.11 (−0.32, 0.10)	0.06 (−0.38, 0.49)	0.115

*CI* confidence interval, *LTFJ* lateral tibio-femoral joint (includes 5 subregions), *MTFJ* medial tibio-femoral joint (includes 5 subregions), *PFJ* patello-femoral joint (includes 4 subregions). Possible score range for bone marrow lesion is 0–3 for each subregion at baseline, 3-, 6- and 12-month

*Statistically significant at *p* ≤ 0.05

## Discussion

In this post-hoc analysis of the effects of sprifermin on cartilage and non-cartilaginous joint tissues, the most notable finding was that cartilage surface morphology showed less worsening from baseline to 12 months in the PFJ, and that BMLs analyzed for the whole knee showed improvement after 100 μg sprifermin treatment from 6 to 12, but not from baseline to 6 months. For other parameters, subregional analyses and observation intervals, the results were not significant, ─ possibly a consequence of small numbers, which make statistical interpretation challenging.

In light of the previous trials showing more marked treatment effects on cartilage in the lateral knee compartment as compared to the medial knee compartment [15, 16], it is interesting to note that in this analysis using subregional analytic approaches, a non-significant tendency toward less worsening in the lateral femorotibial compartment (LTFC) as compared to the medial femorotibial compartment (MTFC) was observed. The reasons for the seemingly preferential effect on the lateral knee compartment in the present and previous studies are still under debate. Commonly, the medial compartment is subjected to more loading and is also more severely affected by OA. Consequently, an anabolic agent acting on cartilage may be less effective in areas of mechanical challenge that are already severely damaged, such as the medial compartment.

A strong association between BMLs and cartilage surface morphology has been shown in previous work using subregional semi-quantitative approaches [9, 17]. While no definitive effects on BML change were seen from baseline to 12 months, more improvement of BMLs was shown from 6 to 12 months for the whole knee analysis, a finding that is not easily explained. Whether cartilage alterations may have secondary biomechanical effects on the adjacent subchondral bone, which may lead to a decrease in localized loading and regression of BMLs remains to be shown [18]. Fluctuations of BMLs have been observed, focusing attention on BMLs as potential treatment targets [3, 16]. The finding that sprifermin may have indirect or direct effects on the subchondral bone hence warrants further exploration.

Finally, no significant changes between treatment and placebo groups were seen for other joint parameters including osteophytes, meniscal pathology, effusion, and synovitis. This may be explained by the pharmacologic mode of action of sprifermin targeting cartilage, the slow course of the disease, and the limited number of patients in each cohort.

Limitations of our post-hoc analysis include the small sample size, and therefore corresponding lack of multiple adjustment in addition to not handling of missing data. Further, we only used matched, rather than all, placebo patients for comparison with the 100 μg cohort, because this was an ascending dose escalation safety study, and in which each of the 10, 30 and 100 μg cohort were randomized and matched with a placebo control at different times throughout the study.

## Conclusions

In summary, positive effects on the patello-femoral cartilage from baseline to 12 months and on BMLs in whole knee analyses were seen from 6 to 12 months. Continued clinical and basic studies are warranted to understand better the potential effects of sprifermin on different joint structures.

## Abbreviations

ANCOVA, analysis of covariance; BMI, body mass index; BMLs, bone marrow lesions; CI, confidence interval; KL, Kellgren-Lawrence; LTFC, lateral femorotibial compartment; LTFJ, lateral tibio-femoral joint; MRI, magnetic resonance imaging; MTFC, medial tibiofemoral compartment; MTFJ, medial tibio-femoral joint; OA, osteoarthritis; PFJ, patello-femoral joint; qMRI, quantitative MRI; SD, standard deviation; sqMRI, semi-quantitative MRI; WORMS, whole-organ magnetic resonance imaging score

## References

[1] Pavelká K, Gatterová J, Olejarová M, Machacek S, Giacovelli G, Rovati LC (2002). Glucosamine sulfate use and delay of progression of knee osteoarthritis: a 3-year, randomized, placebo-controlled, double-blind study. Arch Intern Med.

[2] Brandt KD, Mazzuca SA, Katz BP, Lane KA, Buckwalter KA, Yocum DE (2005). Effects of doxycycline on progression of osteoarthritis: results of a randomized, placebo-controlled, double-blind trial. Arthritis Rheum.

[3] Reginster JY, Badurski J, Bellamy N, Bensen W, Chapurlat R, Chevalier X (2013). Efficacy and safety of strontium ranelate in the treatment of knee osteoarthritis: results of a double-blind, randomised placebo-controlled trial. Ann Rheum Dis.

[4] le Graverand MP H, Clemmer RS, Redifer P, Brunell RM, Hayes CW, Brandt KD (2013). A 2-year randomised, double-blind, placebo-controlled, multicentre study of oral selective iNOS inhibitor, cindunistat (SD-6010), in patients with symptomatic osteoarthritis of the knee. Ann Rheum Dis.

[5] Hunter DJ (2011). Pharmacologic therapy for osteoarthritis-the era of disease modification. Nat Rev Rheumatol.

[6] Ellsworth JL, Berry J, Bukowski T, Claus J, Feldhaus A, Holderman S (2002). Fibroblast growth factor-18 is a trophic factor for mature chondrocytes and their progenitors. Osteoarthritis Cartilage.

[7] Moore EE, Bendele AM, Thompson DL, Littau A, Waggie KS, Reardon B (2005). Fibroblast growth factor-18 stimulates chondrogenesis and cartilage repair in a rat model of injury-induced osteoarthritis. Osteoarthritis Cartilage.

[8] Lohmander LS, Hellot S, Dreher D, Krantz EF, Kruger DS, Guermazi A (2014). Intraarticular sprifermin (recombinant human fibroblast growth factor 18) in knee osteoarthritis: a randomized, double-blind, placebo-controlled trial. Arthritis Rheumatol.

[9] Roemer FW, Felson DT, Wang K, Crema MD, Neogi T, Zhang Y (2013). Co-localisation of non-cartilaginous articular pathology increases risk of cartilage loss in the tibiofemoral joint--the MOST study. Ann Rheum Dis.

[10] Kellgren JH, Lawrence JS (1957). Radiological assessment of osteo-arthrosis. Ann Rheum Dis.

[11] Eckstein F, Wirth W, Guermazi A, Maschek S, Aydemir A (2015). Intra-articular sprifermin not only increases cartilage thickness, but also reduces cartilage loss - location-independent post hoc analysis using MR imaging. Arthritis Rheumatol.

[12] Peterfy CG, Guermazi A, Zaim S, Tirman PF, Miaux Y, White D (2004). Whole-Organ Magnetic Resonance Imaging Score (WORMS) of the knee in osteoarthritis. Osteoarthritis Cartilage.

[13] Hunter DJ, Zhang YQ, Niu JB, Tu X, Amin S, Clancy M (2006). The association of meniscal pathologic changes with cartilage loss in symptomatic knee osteoarthritis. Arthritis Rheum.

[14] Hunter DJ, Zhang YQ, Tu X, Lavalley M, Niu JB, Amin S (2006). Change in joint space width: hyaline articular cartilage loss or alteration in meniscus?. Arthritis Rheum.

[15] Raynauld JP, Martel-Pelletier J, Bias P, Laufer S, Haraoui B, Choquette D (2009). Protective effects of licofelone, a 5-lipoxygenase and cyclo-oxygenase inhibitor, versus naproxen on cartilage loss in knee osteoarthritis: a first multicentre clinical trial using quantitative MRI. Ann Rheum Dis.

[16] Wildi LM, Raynauld JP, Martel-Pelletier J, Beaulieu A, Bessette L, Morin F (2011). Chondroitin sulphate reduces both cartilage volume loss and bone marrow lesions in knee osteoarthritis patients starting as early as 6 months after initiation of therapy: a randomised, double-blind, placebo-controlled pilot study using MRI. Ann Rheum Dis.

[17] Felson DT (2013). Osteoarthritis as a disease of mechanics. Osteoarthritis Cartilage.

[18] Roemer FW, Guermazi A, Javaid MK, Lynch JA, Niu J, Zhang Y (2009). Change in MRI-Detected subchondral bone marrow lesions is associated with cartilage loss: the MOST Study. A longitudinal multicenter study of knee osteoarthritis. Ann Rheum Dis.

